# Reinvestigation of 4-methyl­anilinium dihydrogen phosphite

**DOI:** 10.1107/S1600536810017563

**Published:** 2010-05-19

**Authors:** Karla Fejfarová, Markéta Jarošová, Ilhame Halime, Mohammed Lachkar, Brahim El Bali

**Affiliations:** a Institute of Physics, Na Slovance 2, 182 21 Praha 8, Czech Republic; b Laboratoire d’Ingénierie des Matériaux Organométalliques et Moléculaires, Département de Chimie, Faculté des Sciences, Université Sidi Mohamed Ben Abdellah, BP 1796 (Atlas), 30000 Fès, Morocco; c Department of Chemistry, Faculty of Sciences, University Mohammed 1st, PO Box 717, 60000 Oujda, Morocco

## Abstract

The crystal structure of the title compound, C_7_H_10_N^+^·H_2_PO_3_^−^, has been reported previously by Sabounchei & Naghipour [*Asian J. Chem.* (2003) ▶, **15**, 1677–1686]. A new look at this compound has revealed doubling of the unit cell. The asymmetric unit consists of two 4-methyl­anilinium cations and two dihydrogen phosphite anions. The crystal structure is built upon alternating layers of organic cations and dihydrogen phosphite anions stacked along *c*. The organic layer is stabilized by C—H⋯π interactions. Weak aromatic π–π stacking interactions with centroid–centroid distances of 4.6147 (12), 4.6917 (12), 4.6932 (13) and 4.8366 (13) Å are also observed in the structure. The dihydrogen phosphite anions are linked by O—H⋯O hydrogen bonds into chains running parallel to the *a*-axis direction. These chains are connected to the cation layer by N—H⋯O hydrogen bonds.

## Related literature

For the previously reported structure determination of the title compound, see: Sabounchei & Naghipour (2003 ▶).
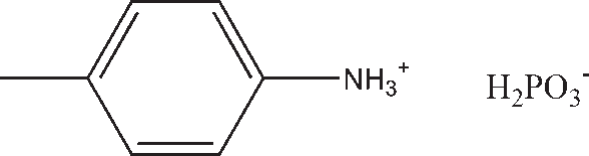


## Experimental

### 

#### Crystal data

C_7_H_10_N^+^·H_2_PO_3_^−^
*M* *_r_* = 189.15
Triclinic, 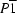

*a* = 9.3053 (7) Å
*b* = 9.4087 (7) Å
*c* = 11.3432 (8) Å
α = 70.253 (7)°
β = 76.304 (6)°
γ = 82.771 (6)°
*V* = 906.99 (12) Å^3^
*Z* = 4
Mo *K*α radiation
μ = 0.27 mm^−1^
*T* = 120 K
0.54 × 0.20 × 0.10 mm

#### Data collection

Oxford Diffraction XCalibur 2 with area-detector Sapphire 2 diffractometer
Absorption correction: multi-scan (*CrysAlis RED*; Oxford Diffraction, 2008 ▶) *T*_min_ = 0.885, *T*_max_ = 0.973
11211 measured reflections
3787 independent reflections
1948 reflections with *I* > 3σ(*I*)
*R*_int_ = 0.036

#### Refinement

*R*[*F* ^2^ > 2σ(*F* ^2^)] = 0.031
*wR*(*F* ^2^) = 0.085
*S* = 0.94
3787 reflections
229 parameters
2 restraints
H atoms treated by a mixture of independent and constrained refinement
Δρ_max_ = 0.18 e Å^−3^
Δρ_min_ = −0.21 e Å^−3^

### 

Data collection: *CrysAlis CCD* (Oxford Diffraction, 2006 ▶); cell refinement: *CrysAlis RED* (Oxford Diffraction, 2008 ▶); data reduction: *CrysAlis RED*; program(s) used to solve structure: *SIR2002* (Burla *et al.*, 2003 ▶); program(s) used to refine structure: *JANA2006* (Petříček *et al.*, 2010 ▶); molecular graphics: *DIAMOND* (Brandenburg & Putz, 2005 ▶); software used to prepare material for publication: *JANA2006*.

## Supplementary Material

Crystal structure: contains datablocks global, I. DOI: 10.1107/S1600536810017563/fk2019sup1.cif

Structure factors: contains datablocks I. DOI: 10.1107/S1600536810017563/fk2019Isup2.hkl

Additional supplementary materials:  crystallographic information; 3D view; checkCIF report

## Figures and Tables

**Table 1:** Hydrogen-bond geometry (Å, °) *Cg*1 and *Cg*2 are the centroids of the C1–C6 and C8–C13 rings, respectively.

*D*—H⋯*A*	*D*—H	H⋯*A*	*D*⋯*A*	*D*—H⋯*A*
N1—H1*a*⋯O6	0.87	1.90	2.760 (2)	167
N1—H1*b*⋯O2^i^	0.87	2.00	2.850 (2)	165
N1—H1*c*⋯O5^ii^	0.87	1.93	2.784 (2)	169
O1—H1*o*⋯O6	0.82 (2)	1.703 (19)	2.523 (2)	173 (3)
N2—H2*a*⋯O5^i^	0.87	2.03	2.875 (2)	165
N2—H2*b*⋯O2^i^	0.87	1.87	2.729 (2)	168
N2—H2*c*⋯O3	0.87	1.91	2.773 (2)	173
O4—H4*o*⋯O3^iii^	0.818 (18)	1.704 (17)	2.516 (2)	171 (2)
C3—H3⋯*Cg*2^iii^	0.96	2.87	3.502 (2)	125
C6—H6⋯*Cg*2	0.96	2.97	3.589 (2)	123
C10—H10⋯*Cg*1^iv^	0.96	2.93	3.655 (2)	133

Symmetry codes: (i) 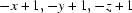; (ii) 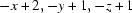; (iii) 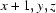; (iv) 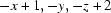.

**Fig. 1.**
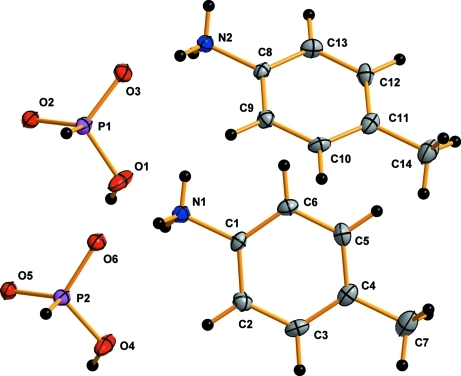
: The molecular structure of C7H10N+ H2PO3-. Displacement ellipsoids are drawn at the 50% probability level.

**Fig. 2.**
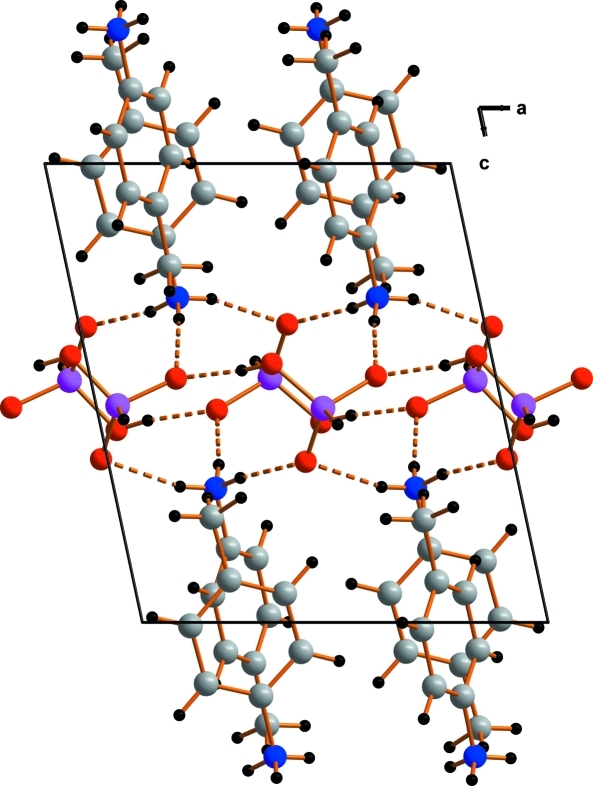
: Crystal packing of title coumpound viewed along the b axis. Hydrogen bonds are displayed as dashed lines. Color code: Grey balls (C), blue balls (N), red balls (O), pink balls (P), black balls (H).

## supplementary crystallographic information

### Experimental

Crystals of the title compound were obtained unintentionally in an attempt to prepare a Ni based hybrid organic-inorganic phosphite. In fact, reactants NiCl_2_ (0.078 g, 5 mmol), H_3_PO_3_ (0.0834 g, 1 mmol) and four drops of *p*-Toluidine were added to 10 ml of distilled water. The solution was heated for 3 hours at 330 K and the resulting greenish solution was left at room temperature. After two weeks, colourless irregular shaped crystals deposited. They were filtered off and washed with a solution of ethanol–water (4:1 v/v). The chemical composition of the reported compound was confirmed by microprobe analysis.

### Refinement

All the hydrogens were discernible in difference Fourier maps and could be refined to reasonable geometry. According to common practise hydrogens attached to C and N atoms were nevertheless kept in ideal positions during the refinement. The O—H distances were restrained to 0.82 Å with σ 0.01. The isotropic temperature parameters of hydrogen atoms were evaluated as 1.2*U_eq_ of the parent atom.

### Crystal data

**Table:**

C_7_H_10_N^+^·H_2_PO_3_^−^	*Z* = 4
*M**_r_* = 189.15	*F*(000) = 400
Triclinic, *P*1	*D*_x_ = 1.385 Mg m^−^^3^
Hall symbol: -P 1	Mo *K*α radiation, λ = 0.71069 Å
*a* = 9.3053 (7) Å	Cell parameters from 1617 reflections
*b* = 9.4087 (7) Å	θ = 3.0–26.5°
*c* = 11.3432 (8) Å	µ = 0.27 mm^−^^1^
α = 70.253 (7)°	*T* = 120 K
β = 76.304 (6)°	Prism, colorless
γ = 82.771 (6)°	0.54 × 0.20 × 0.10 mm
*V* = 906.99 (12) Å^3^	

### Data collection

**Table:**

Oxford Diffraction XCalibur 2 with area-detector Sapphire 2 diffractometer	3787 independent reflections
Radiation source: X-ray tube	1948 reflections with *I* > 3σ(*I*)
graphite	*R*_int_ = 0.036
Detector resolution: 8.3438 pixels mm^-1^	θ_max_ = 26.6°, θ_min_ = 3°
Rotation method data acquisition using ω scans	*h* = −11→11
Absorption correction: multi-scan (*CrysAlis RED*; Oxford Diffraction, 2008)	*k* = −11→11
*T*_min_ = 0.885, *T*_max_ = 0.973	*l* = −14→14
11211 measured reflections	

### Refinement

**Table:**

Refinement on *F*^2^	84 constraints
*R*[*F*^2^ > 2σ(*F*^2^)] = 0.031	H atoms treated by a mixture of independent and constrained refinement
*wR*(*F*^2^) = 0.085	Weighting scheme based on measured s.u.'s *w* = 1/[σ^2^(*I*) + 0.0016*I*^2^]
*S* = 0.94	(Δ/σ)_max_ = 0.006
3787 reflections	Δρ_max_ = 0.18 e Å^−^^3^
229 parameters	Δρ_min_ = −0.21 e Å^−^^3^
2 restraints	

### Special details

**Table:**

Experimental. CrysAlis RED, Oxford Diffraction (2008). Empirical absorption correction using spherical harmonics, implemented in SCALE3 ABSPACK scaling algorithm.
Refinement. The refinement was carried out against all reflections. The conventional *R*-factor is always based on *F*. The goodness of fit as well as the weighted *R*-factor are based on *F* and *F*^2^ for refinement carried out on *F* and *F*^2^, respectively. The threshold expression is used only for calculating *R*-factors etc. and it is not relevant to the choice of reflections for refinement.The program used for refinement, Jana2006, uses the weighting scheme based on the experimental expectations, see _refine_ls_weighting_details, that does not force *S* to be one. Therefore the values of *S* are usually larger then the ones from the SHELX program.

### Fractional atomic coordinates and isotropic or equivalent isotropic displacement parameters (Å^2^)

**Table:**

	*x*	*y*	*z*	*U*_iso_*/*U*_eq_	
P1	0.44268 (6)	0.24716 (7)	0.46676 (6)	0.0165 (2)	
P2	0.94726 (6)	0.25421 (7)	0.47260 (6)	0.0167 (2)	
O1	0.54591 (17)	0.17576 (19)	0.56371 (16)	0.0298 (7)	
O2	0.51328 (15)	0.37070 (16)	0.35039 (13)	0.0195 (6)	
O3	0.29515 (15)	0.29375 (16)	0.53675 (13)	0.0195 (6)	
O4	1.03762 (17)	0.2061 (2)	0.58040 (15)	0.0308 (7)	
O5	1.01622 (15)	0.37753 (16)	0.35577 (13)	0.0184 (6)	
O6	0.78923 (15)	0.28969 (16)	0.53163 (13)	0.0190 (6)	
N1	0.74334 (19)	0.44279 (19)	0.70739 (16)	0.0171 (7)	
N2	0.25053 (18)	0.4549 (2)	0.70792 (16)	0.0168 (7)	
C1	0.7462 (2)	0.3513 (2)	0.8403 (2)	0.0159 (9)	
C2	0.8748 (2)	0.2693 (2)	0.8666 (2)	0.0194 (8)	
C3	0.8833 (2)	0.1901 (2)	0.9927 (2)	0.0203 (9)	
C4	0.7635 (3)	0.1912 (3)	1.0925 (2)	0.0194 (9)	
C5	0.6343 (2)	0.2725 (2)	1.0629 (2)	0.0206 (9)	
C6	0.6246 (2)	0.3533 (2)	0.93645 (19)	0.0179 (8)	
C7	0.7744 (3)	0.1095 (3)	1.2295 (2)	0.0330 (11)	
C8	0.2503 (2)	0.3567 (2)	0.8389 (2)	0.0144 (9)	
C9	0.3142 (2)	0.2119 (2)	0.86021 (19)	0.0173 (8)	
C10	0.3151 (2)	0.1208 (2)	0.98476 (19)	0.0187 (8)	
C11	0.2530 (2)	0.1712 (3)	1.0889 (2)	0.0193 (9)	
C12	0.1901 (2)	0.3180 (2)	1.0644 (2)	0.0193 (8)	
C13	0.1881 (2)	0.4110 (2)	0.94022 (19)	0.0190 (8)	
C14	0.2534 (3)	0.0697 (3)	1.2231 (2)	0.0309 (10)	
H1o	0.6230 (17)	0.219 (3)	0.549 (2)	0.0358*	
H4o	1.1189 (15)	0.242 (3)	0.560 (2)	0.037*	
H1a	0.750815	0.383602	0.661368	0.0205*	
H1b	0.660389	0.496818	0.704816	0.0205*	
H1c	0.817088	0.502228	0.677138	0.0205*	
H2a	0.163763	0.500864	0.70399	0.0202*	
H2b	0.317161	0.52158	0.685108	0.0202*	
H2c	0.270786	0.400831	0.656644	0.0202*	
H2	0.957885	0.267015	0.798343	0.0233*	
H3	0.973162	0.133701	1.011183	0.0244*	
H5	0.550038	0.273093	1.130628	0.0247*	
H6	0.534789	0.409164	0.916969	0.0215*	
H7a	0.864717	0.047813	1.231763	0.0396*	
H7b	0.773841	0.181898	1.27231	0.0396*	
H7c	0.691539	0.046706	1.271737	0.0396*	
H9	0.35734	0.175038	0.789684	0.0207*	
H10	0.3597	0.020317	0.999763	0.0225*	
H12	0.147349	0.355424	1.134695	0.0232*	
H13	0.144188	0.511853	0.924573	0.0228*	
H14a	0.240884	0.129949	1.278716	0.0371*	
H14b	0.173647	0.002559	1.251293	0.0371*	
H14c	0.345892	0.011813	1.225366	0.0371*	
H1p	0.415 (2)	0.132 (2)	0.4315 (18)	0.0198*	
H2p	0.940 (2)	0.131 (2)	0.4421 (18)	0.02*	

### Atomic displacement parameters (Å^2^)

**Table:**

	*U*^11^	*U*^22^	*U*^33^	*U*^12^	*U*^13^	*U*^23^
P1	0.0147 (3)	0.0167 (3)	0.0165 (3)	−0.0020 (3)	−0.0049 (3)	−0.0019 (3)
P2	0.0155 (3)	0.0169 (3)	0.0157 (3)	−0.0013 (3)	−0.0045 (3)	−0.0017 (3)
O1	0.0185 (9)	0.0311 (11)	0.0295 (10)	−0.0094 (8)	−0.0128 (8)	0.0118 (8)
O2	0.0173 (8)	0.0213 (9)	0.0168 (9)	−0.0009 (7)	−0.0046 (7)	−0.0014 (7)
O3	0.0157 (8)	0.0246 (9)	0.0170 (8)	−0.0036 (7)	−0.0025 (7)	−0.0048 (7)
O4	0.0165 (9)	0.0450 (12)	0.0203 (9)	−0.0103 (8)	−0.0072 (8)	0.0086 (9)
O5	0.0173 (8)	0.0204 (9)	0.0144 (8)	−0.0019 (7)	−0.0037 (7)	−0.0011 (7)
O6	0.0148 (8)	0.0219 (9)	0.0178 (8)	−0.0034 (7)	−0.0043 (7)	−0.0017 (7)
N1	0.0156 (10)	0.0174 (10)	0.0163 (10)	−0.0015 (9)	−0.0038 (8)	−0.0024 (9)
N2	0.0153 (10)	0.0174 (10)	0.0154 (10)	−0.0007 (9)	−0.0033 (8)	−0.0021 (8)
C1	0.0195 (13)	0.0138 (13)	0.0145 (12)	−0.0040 (11)	−0.0057 (10)	−0.0019 (11)
C2	0.0164 (12)	0.0218 (12)	0.0198 (12)	−0.0034 (10)	−0.0022 (9)	−0.0064 (10)
C3	0.0198 (12)	0.0158 (12)	0.0269 (13)	0.0013 (10)	−0.0118 (10)	−0.0048 (10)
C4	0.0260 (13)	0.0152 (12)	0.0193 (13)	−0.0042 (10)	−0.0097 (11)	−0.0039 (10)
C5	0.0223 (13)	0.0227 (12)	0.0160 (12)	−0.0044 (10)	0.0001 (10)	−0.0065 (10)
C6	0.0164 (11)	0.0169 (11)	0.0214 (12)	−0.0007 (10)	−0.0071 (9)	−0.0051 (10)
C7	0.0473 (17)	0.0282 (15)	0.0243 (14)	0.0008 (13)	−0.0142 (13)	−0.0059 (12)
C8	0.0131 (12)	0.0159 (13)	0.0130 (12)	−0.0035 (10)	−0.0042 (10)	−0.0011 (10)
C9	0.0175 (12)	0.0193 (12)	0.0166 (11)	−0.0014 (10)	−0.0047 (9)	−0.0067 (9)
C10	0.0181 (12)	0.0131 (11)	0.0243 (12)	0.0006 (9)	−0.0084 (10)	−0.0028 (10)
C11	0.0185 (12)	0.0208 (13)	0.0174 (12)	−0.0078 (11)	−0.0059 (10)	−0.0005 (10)
C12	0.0217 (12)	0.0230 (13)	0.0149 (11)	−0.0057 (10)	−0.0029 (9)	−0.0074 (10)
C13	0.0190 (12)	0.0132 (11)	0.0245 (12)	−0.0019 (9)	−0.0050 (10)	−0.0050 (10)
C14	0.0360 (16)	0.0311 (15)	0.0209 (13)	−0.0043 (12)	−0.0088 (12)	0.0006 (12)

### Geometric parameters (Å, °)

**Table:**

P1—O1	1.5563 (18)	C3—H3	0.96
P1—O2	1.5066 (13)	C4—C5	1.389 (3)
P1—O3	1.5082 (14)	C4—C7	1.504 (3)
P1—H1p	1.35 (2)	C5—C6	1.397 (3)
P2—O4	1.5570 (19)	C5—H5	0.96
P2—O5	1.5033 (13)	C6—H6	0.96
P2—O6	1.5111 (14)	C7—H7a	0.96
P2—H2p	1.33 (2)	C7—H7b	0.96
O1—H1o	0.82 (2)	C7—H7c	0.96
O4—H4o	0.817 (17)	C8—C9	1.382 (3)
N1—C1	1.467 (3)	C8—C13	1.387 (3)
N1—H1a	0.87	C9—C10	1.384 (3)
N1—H1b	0.87	C9—H9	0.96
N1—H1c	0.87	C10—C11	1.393 (3)
N2—C8	1.461 (3)	C10—H10	0.96
N2—H2a	0.87	C11—C12	1.394 (3)
N2—H2b	0.87	C11—C14	1.500 (3)
N2—H2c	0.87	C12—C13	1.388 (3)
C1—C2	1.377 (3)	C12—H12	0.96
C1—C6	1.377 (3)	C13—H13	0.96
C2—C3	1.387 (3)	C14—H14a	0.96
C2—H2	0.96	C14—H14b	0.96
C3—C4	1.390 (3)	C14—H14c	0.96
			
O1—P1—O2	112.94 (9)	C5—C4—C7	120.72 (19)
O1—P1—O3	109.03 (9)	C4—C5—C6	121.28 (19)
O1—P1—H1p	105.0 (8)	C4—C5—H5	119.3623
O2—P1—O3	114.33 (8)	C6—C5—H5	119.3618
O2—P1—H1p	109.7 (7)	C1—C6—C5	118.73 (19)
O3—P1—H1p	105.2 (8)	C1—C6—H6	120.6345
O4—P2—O5	113.03 (9)	C5—C6—H6	120.6348
O4—P2—O6	107.47 (9)	C4—C7—H7a	109.4716
O4—P2—H2p	106.5 (8)	C4—C7—H7b	109.4709
O5—P2—O6	114.87 (8)	C4—C7—H7c	109.4717
O5—P2—H2p	109.9 (7)	H7a—C7—H7b	109.4704
O6—P2—H2p	104.4 (8)	H7a—C7—H7c	109.4711
P1—O1—H1o	115.9 (15)	H7b—C7—H7c	109.4716
P2—O4—H4o	114.8 (16)	N2—C8—C9	119.6 (2)
C1—N1—H1a	109.4712	N2—C8—C13	119.48 (18)
C1—N1—H1b	109.4716	C9—C8—C13	120.91 (19)
C1—N1—H1c	109.4717	C8—C9—C10	119.0 (2)
H1a—N1—H1b	109.4701	C8—C9—H9	120.5149
H1a—N1—H1c	109.4712	C10—C9—H9	120.5151
H1b—N1—H1c	109.4716	C9—C10—C11	121.74 (19)
C8—N2—H2a	109.471	C9—C10—H10	119.1322
C8—N2—H2b	109.4707	C11—C10—H10	119.1326
C8—N2—H2c	109.4708	C10—C11—C12	118.00 (19)
H2a—N2—H2b	109.4716	C10—C11—C14	120.8 (2)
H2a—N2—H2c	109.4717	C12—C11—C14	121.2 (2)
H2b—N2—H2c	109.4715	C11—C12—C13	121.1 (2)
N1—C1—C2	118.50 (17)	C11—C12—H12	119.4449
N1—C1—C6	120.20 (18)	C13—C12—H12	119.4445
C2—C1—C6	121.24 (19)	C8—C13—C12	119.27 (19)
C1—C2—C3	119.45 (19)	C8—C13—H13	120.366
C1—C2—H2	120.2763	C12—C13—H13	120.3658
C3—C2—H2	120.2765	C11—C14—H14a	109.4716
C2—C3—C4	121.0 (2)	C11—C14—H14b	109.4705
C2—C3—H3	119.5091	C11—C14—H14c	109.471
C4—C3—H3	119.5082	H14a—C14—H14b	109.4714
C3—C4—C5	118.3 (2)	H14a—C14—H14c	109.4717
C3—C4—C7	121.0 (2)	H14b—C14—H14c	109.4711
			
N1—C1—C2—C3	175.68 (18)	N2—C8—C9—C10	179.07 (18)
C6—C1—C2—C3	−1.6 (3)	C13—C8—C9—C10	0.2 (3)
N1—C1—C6—C5	−175.96 (18)	N2—C8—C13—C12	−179.06 (18)
C2—C1—C6—C5	1.3 (3)	C9—C8—C13—C12	−0.2 (3)
C1—C2—C3—C4	0.7 (3)	C8—C9—C10—C11	0.2 (3)
C2—C3—C4—C5	0.6 (4)	C9—C10—C11—C12	−0.5 (3)
C2—C3—C4—C7	−178.0 (2)	C9—C10—C11—C14	179.1 (2)
C3—C4—C5—C6	−0.9 (4)	C10—C11—C12—C13	0.5 (3)
C7—C4—C5—C6	177.7 (2)	C14—C11—C12—C13	−179.1 (2)
C4—C5—C6—C1	0.0 (3)	C11—C12—C13—C8	−0.2 (3)

### Hydrogen-bond geometry (Å, °)

**Table:**

Cg1 and Cg2 are the centroids of the C1–C6 and C8–C13 rings, respectively.

**Table:**

*D*—H···*A*	*D*—H	H···*A*	*D*···*A*	*D*—H···*A*
N1—H1a···O6	0.87	1.90	2.760 (2)	167
N1—H1b···O2^i^	0.87	2.00	2.850 (2)	165
N1—H1c···O5^ii^	0.87	1.93	2.784 (2)	169
O1—H1o···O6	0.82 (2)	1.703 (19)	2.523 (2)	173 (3)
N2—H2a···O5^i^	0.87	2.03	2.875 (2)	165
N2—H2b···O2^i^	0.87	1.87	2.729 (2)	168
N2—H2c···O3	0.87	1.91	2.773 (2)	173
O4—H4o···O3^iii^	0.818 (18)	1.704 (17)	2.516 (2)	171 (2)
C3—H3···Cg2^iii^	0.96	2.87	3.502 (2)	125
C6—H6···Cg2	0.96	2.97	3.589 (2)	123
C10—H10···Cg1^iv^	0.96	2.93	3.655 (2)	133

Symmetry codes: (i) −*x*+1, −*y*+1, −*z*+1; (ii) −*x*+2, −*y*+1, −*z*+1; (iii) *x*+1, *y*, *z*; (iv) −*x*+1, −*y*, −*z*+2.
